# Response of a New Low-Coherence Fabry-Perot Sensor to Hematocrit Levels in Human Blood

**DOI:** 10.3390/s140406965

**Published:** 2014-04-21

**Authors:** Małgorzata Jędrzejewska-Szczerska

**Affiliations:** Faculty of Electronics, Telecommunications and Informatics, Department of Metrology and Optoelectronics, Gdańsk University of Technology, 11/12 Narutowicza Street, Gdańsk 80-233, Poland; E-Mail: mjedrzej@eti.pg.gda.pl; Tel.: + 48-583-471-361

**Keywords:** low-coherence interferometer, Fabry-Perot interferometer, fiber optic sensors, hematocrit

## Abstract

In this paper, a low-coherence Fabry-Perot sensor with a spectrally measured signal processing response to the refractive index of liquids is presented. Optical fiber sensors are potentially capable of continuous measuring hematocrit levels in blood. Low-coherence Fabry-Perot interferometric sensors offer a robust solution, where information about the measurand is encoded in the full spectrum of light reflected from the sensing interferometer. The first step in the research on such sensor is the assessment of its performance under favorable conditions, *i.e.*, using blood samples from healthy volunteers tested *in vitro*. Such an experiment was conducted using a sensor comprising a superluminescent diode source, an optical spectrum analyzer working as the detection setup and a sensing Fabry-Perot interferometer providing high interference contrast. The response of this sensor was recorded for several samples and compared with the reference laboratory method. The coefficient of determination (R^2^) for a linear relationship between the results given by both methods was 0.978 and the difference between these results was less than 1%. The presented results suggest that further research into the performance of the sensor is merited.

## 1. Introduction

Optical fiber sensors offer several considerable advantages in comparison to their electronic counterparts. They can be manufactured from dielectric materials which make them immune to any electric and magnetic fields used in medical diagnosis and therapy. Using light as a means of carrying information, optical fiber sensors do not emit any electromagnetic noise. They can operate with user-replaceable transducers which can be mass-produced at a low cost. Reducing the size of these transducers, often to less than a hundred micrometers, makes them less invasive to the environment in which they operate and improves their response speed. When information about the value of the quantity measured by the optical fiber sensor is transmitted as a change in phase, wavelength or spectrum, the sensor is immune to any mechanical or acoustical disturbance which may vary only with respect to the intensity of the transmitted signal [1].

Recently, most optoelectronic research in the biomedical area has been focused on spectral measurement techniques [2–4], Optical Coherence Tomography [5,6] and Raman scattering [7,8]. Optical fiber sensors have a considerable potential in biomedical application.

The use of low-coherence optical-fiber Fabry-Perot interferometric sensors [9–12] as sensors of hematocrit levels of whole human blood offers advantages over classical analytical methods, such as: the lack of special preparation of whole human blood samples, the very small amount of blood needed for the measurements, and very short measurement times. In this article, the ability of a low-coherence optical fiber sensor using a Fabry-Perot interferometer with spectral signal processing to assess the hematocrit level in whole human blood without any special preparation under favorable conditions is presented. An optimal interferometer configuration giving a visibility of measured signals close to 1 has been achieved, and a series of measurements of the investigated liquids has been obtained by using this construction.

## 2. Fiber-Optic Fabry-Perot Interferometer

**Figure 1.**
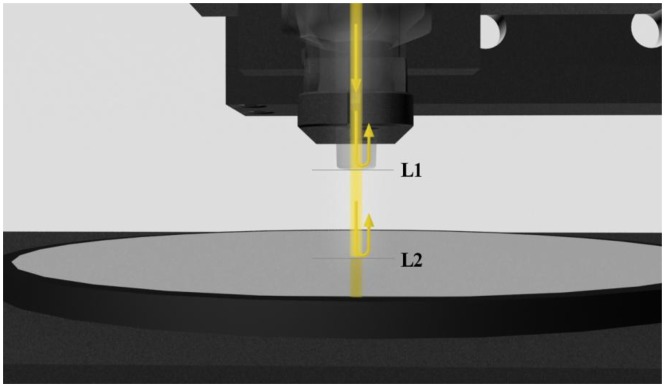
The construction of fiber-optic Fabry-Perot interferometer: L1, L2—the first and second reflective layers, respectively.

The Fabry-Perot interferometer made from bulk optical components consists of two flat transparent plates P1 and P2, parallel to each other and separated by a distance d. Inner surfaces of P1 and P2 are coated with highly reflective layers L1 and L2. Expressions for intensity of light reflected from and transmitted by this interferometer are derived in most cases under three assumptions: (1) the interferometer is illuminated by a plane wave; (2) layers L1 and L2 have the same reflectivity; (3) the interferometer is lossless. Such a derivation yields well-known classic formulas [13]. In contrast, the fiber-optic Fabry-Perot interferometer, shown schematically in Figure 1, is illuminated by a divergent beam from a single-mode fiber. Its reflective layers L1 and L2 may have different reflectivity and may not be parallel [10]. Moreover, the layers and the medium between them may be absorbing. Consequently, the classic formulas do not hold.

In further discussing the fiber-optic Fabry-Perot interferometer, it will be assumed that layers L1 and L2, as well as the medium in the interferometer cavity, are non-absorbing. Reflection coefficients of these layers, defined as the ratios of amplitudes of electric field vectors reflected to incident, are r1 and r2, respectively. Light exiting in the fiber can be described using Gaussian beam formalism [14,15]. The waist of the beam is located at the end surface of the fiber, *i.e.*, in the reflective layer L1. The diameter 2W0 of the beam in the waist is equal to the Mode-Field Diameter MFD of the fiber. The coupling loss coefficient α(x,n) can be defined as:

(1) $$\alpha \left(x,n\right)=\frac{A_{R}\left(x,n\right)}{A_{I}}$$

where *A_I_*—amplitude of the beam incident on the interferometer, *A_R_*—amplitude of the beam coupled back to the fiber, *n*—refractive index of the medium in the interferometer, *x*—distance propagated by the beam in the interferometer. It can be assumed that the coupling loss coefficient α decreases with *x* at the same rate as the amplitude of the Gaussian beam propagating in the interferometer, *i.e.*:

(2) $$\alpha \left(x,n\right)\sim {\left(1+{\left(\frac{x}{x_{0}}\right)}^{2}\right)}^{-\frac{1}{2}}$$

As a result of multiple reflections in the cavity, a series of beams are coupled back to the fiber. Their amplitudes can be expressed as:

(3) $$\begin{array}{ll} A_{1} & =r_{1}A_{I}\\ A_{2} & =r_{2}{\left(1-r_{1}\right)}^{2}\alpha \left(2x_{FP},n\right)A_{I}\\ A_{3} & =r_{2}^{2}r_{1}{\left(1-r_{1}\right)}^{2}\alpha \left(4x_{FP},n\right)A_{I}\\ & \vdots \\ A_{M} & \begin{array}{lll} =r_{2}^{M-1}r_{1}^{M-2}{\left(1-r_{1}\right)}^{2}\alpha \left(2\left(M-1\right)x_{FP},n\right)A_{I} & \text{for} & M\geq 2 \end{array} \end{array}$$

where *A_i_*—amplitude of *i*-th reflected beam, *r_1_, r_2_*—reflection coefficients of *L_1_* and *L_2_* respectively, *α*—coupling loss coefficient, *x_FP_*—length of the Fabry-Perot cavity. Phase difference δ between *i*-th and *i* + *1*-th beam is:

(4) $$\delta =4\pi \frac{nx_{FP}}{\lambda }$$

where λ—wavelength.

The complex amplitude *A_R_* of the sum of the reflected beams is given by:

(5) $$A_{R}=A_{1}+A_{2}e^{-i\delta }+\ldots \ldots ..+A_{N}e^{-iN\delta }$$

where δ—phase difference given by equation 4, *A_N_*—amplitude of *N*-th reflected beam.

Because of the presence of the coupling loss coefficient *α* in *A_i_*, the amplitudes *A_i_* decrease faster than those of the same Fabry-Perot interferometer illuminated by a plane wave. Consequently, the number of beams effectively contributing to the interference is smaller than that in the plane wave-illuminated interferometer case.

## 3. Low-Coherence Fiber-Optic Fabry-Perot Sensor

A Fabry-Perot interferometer designed for investigation of the refractive index of bioliquids should operate in the reflection mode in order to simplify the setup. The Fabry-Perot interferometer is a multibeam interferometer. However, a biosensor for investigation of the refractive index of liquids should be a low-finesse Fabry-Perot interferometer in order to obtain an interferometer with a transfer function as for two-beam interferometers. The optical-fiber Fabry-Perot interferometer has been made with the use of a conventional single mode optical fiber, simplified construction of which is shown in Figure 1.

Its reflective layers have been produced by the boundaries: fiber optic—investigation sample (*L_1_*) (air in Figure 1) and investigation sample (air in Figure 1)—mirror (*L_2_*). It can be noted that any change of the investigated sample causes a change in the reflection coefficient of the Fabry-Perot mirror [14].

Amplitudes A_1_ and A_2_ of the waves reflected from the first and the second surface can be written as:

(6) $$\begin{array}{ll} A_{1} & =r_{1}A_{I}\\ A_{2} & =r_{2}{\left(1-r_{1}\right)}^{2}\alpha \left(2x_{FP},n\right)A_{I} \end{array}$$

where *A_I_*—amplitude of the incident beam; *A_1,2_*—amplitude of the first and the second reflected beam, respectively; *r_1_, r_2_*—reflection coefficients of *L_1_* and *L_2_* respectively, *α*—coupling loss coefficient. The phase difference *δ* between interfering beams can be described by Equation (4)

Optical intensity at the output of such an interferometer can be expressed as [13,14]:

(7) $$I_{\text{out}}=\langle AA*\rangle$$

where *A* = *A_1_* + *A_2_*, *A1* and *A2*—amplitudes of the electric vector of the light wave reflected from the first and the second reflective surfaces inside the sensing interferometer respectively, brackets 〈〉 denote time averages; asterisk * denotes the complex conjugation. Optical intensity at the output of such interferometer can be expressed as [13,14]:

(8) $$I(\lambda )=S(\lambda )\left[1+V_{0}\cos\left(\delta (\lambda )\right)\right]$$

where: *S(λ)*—spectral distribution of the light source; *V_0_*—visibility of measurement signal, δ—phase difference between interfering beams: $\delta (\lambda )=\frac{4\pi \Delta }{\lambda }$ , Δ—optical path difference, where optical path is the product of the values of: geometrical dimension and refractive index of path.

Any change of the optical path difference in Fabry—Perot interferometer causes the change of the frequency modulation of the measured signal spectrum. If δ (λ) = *0*, then there is no spectral modulation. If the phase difference between the interfering beams varies from zero, then the modulation of the measured signal spectrum appears and changes with the change of phase difference—so with the change of the optical path difference in interferometer. The expand measurement theory of low-coherence interferometry with signal procesing in spectral domain was presented in details in [16].

Hence, knowing *I*(*λ*) and having the constant geometrical dimensions of the interferometer cavity it is possible to calculate the refractive index *n* of the medium in the cavity. This signal processing is time-consuming, but it is not sensitive to any transmission changes of the optical system. This is possible because information about the measurand is encoded in the spectra of the measured signal. Therefore, signal processing in the spectral domain can be successfully used in low-coherence sensors.

## 4. Experiments and Results

**Figure 2.**
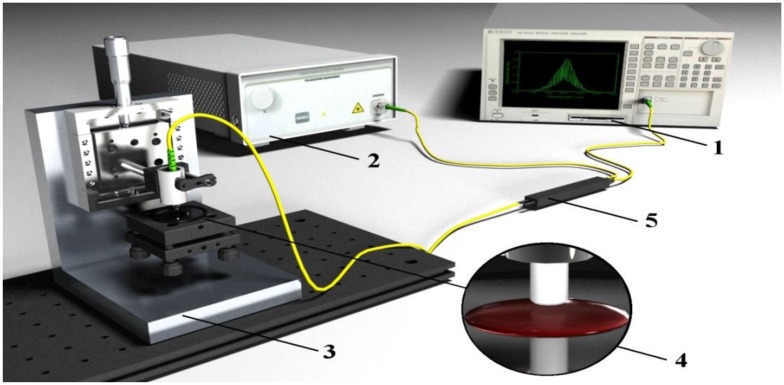
The experimental set-up: (**1**) optical spectrum analyzer; (**2**) superluminescent diode; (**3**) micro-mechanical stage; (**4**) Fabry-Perot interferometer; (**5**) fiber-optic coupler.

An experimental setup was designed and built accordingly to the layout shown in Figure 2. A superluminescent diode (SLED) type S1300-G-I-20 (produced by SUPERLUM, Cork, Ireland) with peak wavelength λ_P_ = 1,290 nm, Gaussian spectral density and Δλ_FWHM_ = 50 nm was used as the broadband source. An Ando AQ6319 optical spectrum analyzer (ANDO (recently Yokogawa Electric Corporation) Tokyo, Japan) with resolution bandwidth set to 1 nm was used in the detection setup. All interconnections, as well as the coupler, were made from standard single-mode telecommunication fiber SMF-28.

The fibre-optic Fabry-Perot interferometer was formed by the uncoated end surface of the single-mode fiber (*r_1_* = 0.2) and the silver mirror (*r_2_* = 0.995) (Figure 1). The fiber is attached to a translation table equipped with a differential adjuster changing the cavity length *x_FP_*.

**Figure 3.**
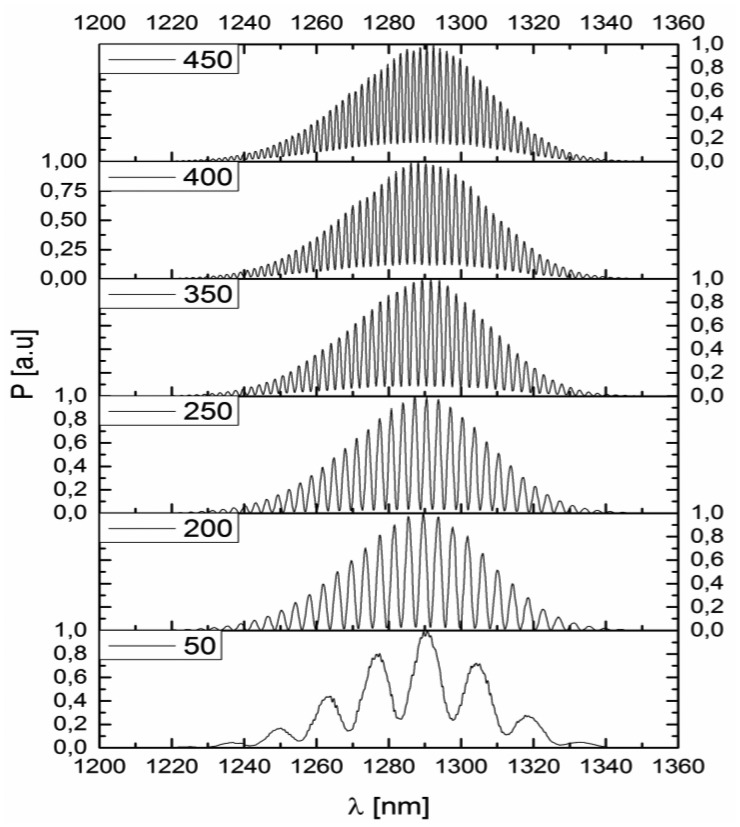
The spectrum of the measured optical signal for the Fabry-Perot cavity of: 450 μm, 400 μm, 350 μm, 250 μm, 200 μm and 50 μm, respectively.

This interferometer was connected to the measurement system. After preliminary testing, the response of the interferometer (*i.e.*, the spectrum acquired by the optical spectrum analyzer) was recorded for several values of cavity length *x_FP_*. The optimum—maximum value of visibility of the measured spectra has been performed for the cavity length of 200 μm, which can be seen in Figure 3. The visibility of the measured signal reaches its maximum (*V_0_* = 0.95) for *x_FP_OPT_* = 200 μm, steadily decreasing for *x_FP_* smaller and greater than *x_FP_OPT_*.

The subsequent experimental process was performed following carefully all of the relevant laboratory procedures, especially controlling the temperature of liquid samples. For each sample under investigation, the response of the Fabry-Perot sensing interferometer was recorded for further analysis. Time required for single measurement was 0.8–1.2 s, because relatively slow optical spectrum analyser was used instead of a dedicated spectroscope. Firstly, a series of samples with a known refractive index (e.g., cyclohexane, methyl salicylate) were performed. More than 300 measurements allowed us to obtain the relationship between the distance between the position of adjacent maximums in the measured signal spectrum and the refractive index of samples. A few samples measured values have been shown in Table1.

**Figure 4.**
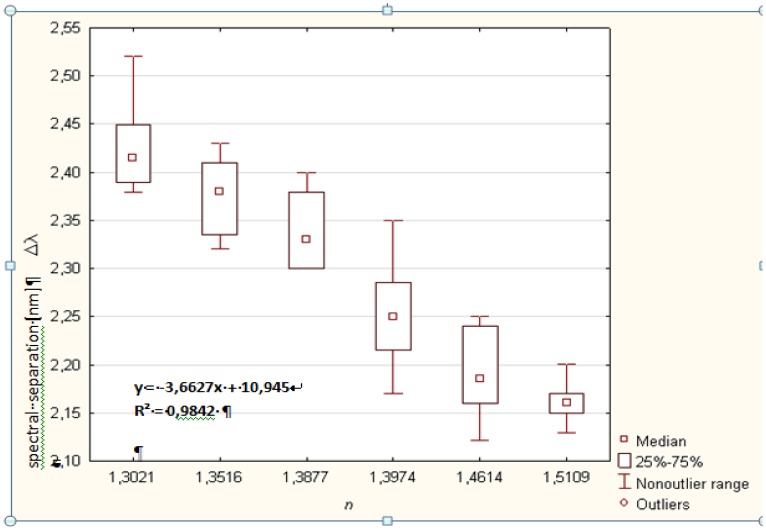
Spectral separation in the spectra of the measured signal *vs.* refractive index.

The relationship between the refractive index and the *spectral separation* is presented in Figure 4. Assuming a linear dependence between these variables, a least-square linear regression was performed, yielding the very high determination coefficient of the measured value R^2^ = 0.984.

**Figure 5.**
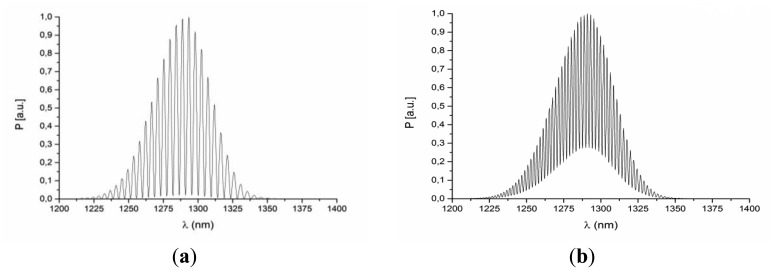
Measured: (**a**) reference signal; (**b**) signal from phosphate buffer saline.

The refractive index of the investigated liquids can be calculated by the use of the calibration curve (Figure 4) [17]. The example spectra corresponding to bioliquids with different value of the refractive index are presented in Figure 5.

The visibility of the interference signal is good enough to confirm the validity of the designed approach. Comparing the spectra from Figure 5a—reference signal and Figure 5b—the measured signal from phosphate buffer saline, it is possible to note the difference between the distance between maximums in the measured signal spectra caused by the change in the refractive index of investigated liquids in the interferometer cavity.

Based on the data presented above, it is safe to conclude that the relationship between the response of the investigated sensor and the refractive index, measured by the standard method, is well defined and the performance of the investigated sensor under favorable experimental conditions, in which it was tested, is adequate at this stage of research.

Investigation of the refractive index of bioliquids, such as whole human blood, is really hard to perform, because of its complex nature. The value of the refractive index of human blood depends on the refractive indices of its various components: blood plasma, proteins, erythrocytes. Furthermore, this value changes with different values of e.g., erythrocytes amount in whole blood. However, a series of 93 samples of 2-mL whole human blood samples were collected by the Gdansk Blood Donor Centre from a wide representative group of rather healthy volunteers. Consequently, the hematocrit level in the samples varied from 30% to 50%. The subsequent experimental process was performed carefully following all of the relevant laboratory procedures, especially controlling the temperature of blood samples.

First, the hematocrit level in each blood sample was assessed by the fiber-optic sensor. Subsequently, the Gdansk Blood Donor Centre performed reference measurement of the hematocrit level in each blood sample using the standard procedure. Both measurements were performed within 24 h from the donation to ensure correct and consistent results. For each blood sample under investigation, the response of the Fabry-Perot sensing interferometer, *i.e.*, the spectrum acquired by the optical spectrum analyzer, was recorded for further analysis.

**Figure 6.**
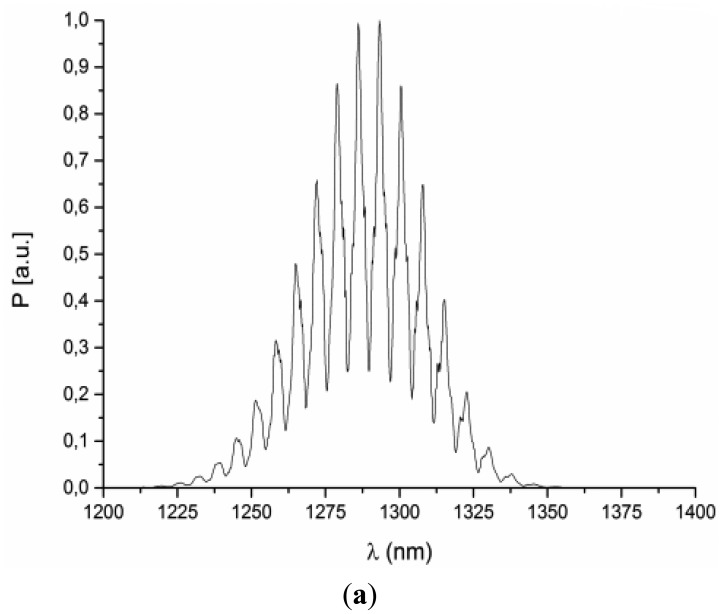

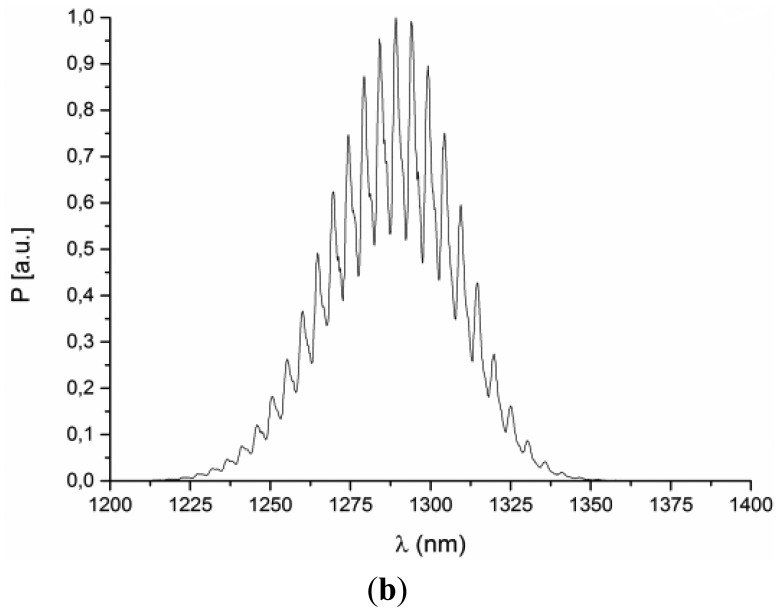
Measured signal from human blood with hematocrit: (**a**) HCT = 38.3%; (**b**) HCT = 40.3%.

**Figure 7.**
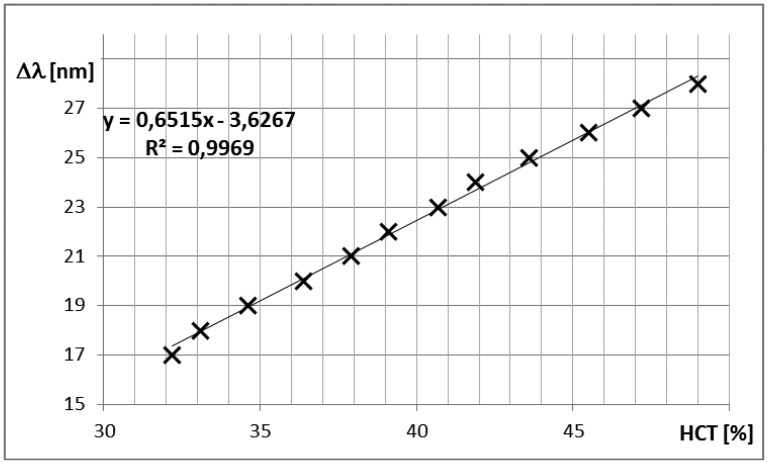
Spectral separation in the spectra of the measured signal *vs.* HCT level.

The example spectra corresponding to blood samples with the hematocrit (HCT) level of 38.3% and 40.4% are presented in Figure 6a,b, respectively. The visibility of the interference signal is good, again confirming the validity of the designed approach. Comparing the spectra from Figure 6, it is possible to note the shift of the fringe pattern caused by the change in the refractive index of blood in the interferometer cavity. The relationship between the hematocrit level and the spectral separation in the central 50 nm range of the acquired spectrum is presented in Figure 7. Assuming linear dependence between these variables, a least-square linear regression was performed, yielding the coefficient of determination R2 = 0.978. Differences between the measured HCT level and the least-square linear model prediction are random and, with one exception (HCT level = 49%), their magnitude is below 1%.

## 5. Discussion

Validation of the sensor carried out with use of set of different liquids and more than 300 measurements allowed us to confirm the unequivocal relationship between the distance between the position of adjacent maxima in the measured spectrum and the refractive index of samples. It was confirmed that the relationship between the refractive index and the *spectral separation* is linear, with a least-square linear regression coefficient R^2^ equal to 0.984.

Validation of the measurements of hematocrit levels in blood was based on assumption that hematocrit changes cause changes in the real part of the refractive index of blood. Experiments using blood samples from healthy volunteers yielded a linear relationship between the sensor's output and the hematocrit level in the range from 30% to 50%. The difference between the linearized sensor output and the hematocrit level measured with the reference method was below 1%, while the determination coefficient of the linear model was quite high (R^2^ = 0.978), which is close to value obtained during the validation of the sensor. Moreover it was confirmed that despite of increase of imaginary part of the refractive index (signal losses) visibility of the fringes in interference spectra is sufficient to determine peak positions and the spectral separation.

Based on the data presented above, it is safe to conclude that the relationship between the response of the investigated sensor and the HCT level, measured by the standard method in healthy persons, is well defined and the performance of the investigated sensor under favorable experimental conditions, in which it was tested, and adequate at this stage of research. This is a necessary condition for further research which will cover three areas: (1) response of the sensor to blood samples with a full range of HCT levels and abnormalities in the blood composition; (2) modelling of the dependence between the response of the sensor and HCT level; and (3) optimization of the sensor ensuring its correct long-term operation and insensitivity to other factors that may affect the measurement results.

## 6. Conclusion

A low-finesse Fabry-Perot interferometer was designed using the method outlined and validated in this paper. Optimized to provide maximum interference contrast and illuminated by a single-mode fiber, the interferometer was used in a fiber optic sensor for assessing hematocrit levels in blood. Such measurements could be performed continuously and repeated every second, if needed. By replacing the thin-film metal mirror in the sensing cavity with a dielectric broadband mirror, it is possible to build the sensing interferometer entirely from dielectric non-toxic materials, allowing it to be used in medical diagnosis, performed even in the presence of strong RF or microwave electromagnetic fields.

Such a sensor is made from dielectric material which makes it immune to electromagnetic fields [20], ionization and chemical environments. For this reason, it can be the only solution for monitoring physiological parameters of patients during medical diagnosis, such as medical examination by the use of nuclear magnetic resonance (NMR) or radiotherapy.

More research is needed in order to ensure the consistent response of the sensor and its insensitivity to other parameters that can influence the measurement process. The long-term stability of the sensor and the improved algorithms of data processing will also be addressed in the future research

## Figures and Tables

**Table 1.** Refractive index results of some investigated bioliquids.

**Investigation Liquid**	**Measured Refractive Index**	**Theoretical Refractive Index**
Glycerol	1.461	1.475 [18]
Phosphate buffer saline	1.326	1.332 [18]
Methyl alcohol	1.319	1.329 [19]
Methyl salicylate	1.51	1.51 [19]
